# The Use and Effectiveness of Triple Multiplex System for Coding Region Single Nucleotide Polymorphism in Mitochondrial DNA Typing of Archaeologically Obtained Human Skeletons from Premodern Joseon Tombs of Korea

**DOI:** 10.1155/2015/850648

**Published:** 2015-08-06

**Authors:** Chang Seok Oh, Soong Deok Lee, Yi-Suk Kim, Dong Hoon Shin

**Affiliations:** ^1^Bioanthropology and Paleopathology Lab, Institute of Forensic Science, Seoul National University College of Medicine, 28 Yongon-dong, Chongno-Gu, Seoul 110-799, Republic of Korea; ^2^Department of Anatomy, Seoul National University College of Medicine, 28 Yongon-dong, Chongno-Gu, Seoul 110-799, Republic of Korea; ^3^Department of Forensic Medicine, Seoul National University College of Medicine, 28 Yongon-dong, Chongno-Gu, Seoul 110-799, Republic of Korea; ^4^Department of Anatomy, Ewha Womans University School of Medicine, 911-1 Mok-6-dong, Yangcheon-Gu, Seoul 158-710, Republic of Korea

## Abstract

Previous study showed that East Asian mtDNA haplogroups, especially those of Koreans, could be successfully assigned by the coupled use of analyses on coding region SNP markers and control region mutation motifs. In this study, we tried to see if the same triple multiplex analysis for coding regions SNPs could be also applicable to ancient samples from East Asia as the complementation for sequence analysis of mtDNA control region. By the study on Joseon skeleton samples, we know that mtDNA haplogroup determined by coding region SNP markers successfully falls within the same haplogroup that sequence analysis on control region can assign. Considering that ancient samples in previous studies make no small number of errors in control region mtDNA sequencing, coding region SNP analysis can be used as good complimentary to the conventional haplogroup determination, especially of archaeological human bone samples buried underground over long periods.

## 1. Introduction

Ancient DNA (aDNA) analysis is very important for understanding the origin and evolution of mankind in history. Of various aDNA studies, analysis on mitochondrial DNA (mtDNA) is one of the best methods to know the phylogeny of archaeologically obtained human samples. mtDNA shows maternal haplotype lineage that is passed down throughout each generation without changing and reshuffling of DNA. In fact, it could be analyzed successfully even in case where nuclear DNA (nDNA) is degraded seriously [1].

Recently, about the determination of East Asian mtDNA haplogroups, Lee et al. [2] showed that Korean mtDNA could be allocated into 15 haplogroups by two different multiplex systems for 21 coding region SNP markers and one deletion motif. As Koreans do have many D4 subhaplogroups, the third set of PCR multiplex systems was also used for defining them in much detail. Authors showed that East Asian mtDNA haplogroups, especially those of Koreans, could be successfully assigned by the multiplex analysis system they developed [2]. As many previously published works exhibited that some of mtDNA data from degraded samples were not sufficiently authentic, the establishment of more tools for detecting possible sequence errors looks valuable to concerned researches.

The multiplex system was originally designed for mtDNA analysis of the degraded samples frequently met in the field of forensic science [2]. However, the technique looks very suggestive to the anthropologists in East Asia as well. Like forensic scientists, the biological anthropologists always tried to analyze the aDNA that is seriously degraded, remaining in archaeological human samples by small amounts. Therefore, sequencing errors in aDNA analysis have always been the researchers' concern. Since archaeological and forensic DNA typing share common subjects to be considered for making their studies on the degraded samples more successful and authentic, many experimental methods developed for forensic science have been also applied to aDNA researches.

In this respect, we wonder if the mtDNA typing by use of coding region SNP analysis could be also applicable to ancient samples from archaeological sites in East Asia. As the technique was proven to be time-, cost-, and target DNA-saving [2], it can be used as a good complimentary to conventional aDNA sequencing if both methods could show well-matching results from archaeologically obtained samples. However, regretfully enough, there were not any previous researches on how perfectly this multiplex system can be applied to archaeologically obtained human samples buried underground for several hundred to thousand years.

For the past several years, we tried to build a skeletal series consisting of human bones collected from 16th to 18th century Joseon tombs in South Korea. Our previous reports on the collection have revealed information concerning the health and disease status of premodern Korean people [3–9]. Using the same human skeleton collection, we undertook the experiments to compare haplogroup-directed data made by two different methods: conventional control region sequencing and analysis of coding region SNP markers. It determines whether the analysis of the triple multiplex system for coding region SNP analysis, like the forensic cases, could be also useful for the mtDNA analysis of hundred-year-old human bones from archaeological sites.

## 2. Materials and Methods

Human skeletons (*n* = 11) collected from 16th to 18th century Korean tombs (Joseon Dynasty) were used in this study. Sex determination was made on the basis of morphological differences manifest in the pelvic bone, by the examination of greater sciatic notch, preauricular sulcus, ischiopubic ramus, subpubic angle, subpubic concavity, and ventral arc [10, 11]. Considered ancillary indicators for sex determination were skull structures, specifically the nuchal crest, the mastoid process, the supraorbital margin, the glabella, and the mental eminence [12, 13]. Age was also estimated by auricular-surface degeneration of the hipbone, based on the degree of transverse organization, granularity, apical activity, retroauricular area degeneration, and auricular-surface porosity [14]. The age was accordingly categorized into eight phases: 1-2, young adult (20–35 years old); 3–6, middle-aged (36–50 years old); and 7-8, old adult (over 50 years old).

The femur fragments from the skeletal remains were used for aDNA analysis in this study. The surfaces of the bones were removed using a sterilized knife, after which they were exposed to UV irradiation for 20 min and subsequently immersed in 5.4% (w/v) sodium hypochlorite. After the samples were washed with distilled water and absolute ethanol, they were air-dried and pulverized to a fine powder using a SPEX 6750 Freezer/Mill (SPEX SamplePrep, Metuchen, NJ) [15, 16]. Bone powder (0.5 g) was incubated in 1 mL of lysis buffer (EDTA 50 mM, pH 8.0; 1 mg/mL of proteinase K; SDS 1%; 0.1 M DTT) at 56°C for 24 h. Total DNA was extracted with an equal volume of phenol/chloroform/isoamyl alcohol (25 : 24 : 1) and then was treated with chloroform/isoamyl alcohol (24 : 1). DNA isolation and purification were performed using a QIAmp PCR purification kit (Qiagen, Hilden, Germany). The purified DNA was eluted in 50 *μ*L of EB buffer (Qiagen) [17–20].

During sampling or lab work, we always wore protection gloves, masks, gowns, and head caps. Our aDNA lab facilities were set up in accordance with the protocol of Hofreiter et al. [21]. The rooms for aDNA extraction or PCR preparation were physically separated from our main PCR lab. The DNA extraction/PCR preparation rooms were equipped with night UV irradiation, isolated ventilation, and a laminated flow hood. The other procedures for authentic aDNA analysis, suggested by Hofreiter et al. [21], were also followed by us.

Three multiplex PCR systems used in this study were originally designed to detect 21 SNPs and a 9-bp deletion motif [2], and the sizes of each PCR amplicon were originally designed below 200 bp, for increasing success yields during DNA typing with degraded samples. By multiplex PCR reactions I and II, major 15 haplogroups could be detected from East Asian samples. Briefly, multiplex I reaction consisted of primers for eight different SNP loci (s4491, s5417, s7642, s8793, s8794, s10397, s10400, and s14668), typing the haplogroups M9, N9, M11, M10, A, D5, M, and D4, respectively. Multiplex II reaction also tested seven SNP loci (s3970, s4833, s4883, s7196, s8281-8289d, s9824, and s12705) for decision of the haplogroups R9, G, D, M8, B, M7, and R, respectively. Haplogroup D4, one of the most frequent haplogroups in East Asian population, was further subdivided into D4, D4a, D4b, D4e, D4g, D4h, and D4j by seven SNP loci (s3010, s14979, s8020, s11215, s8701, s5048, and s11696) of multiplex III reaction [2, 22–25].

Multiplex PCR amplification was done in a 20 *μ*L reaction volume, containing 40 ng of template DNA, Ampli*Taq* Gold 360 Master Mix (Life Technologies, USA), and appropriate concentrations of each primer. Thermal cycling was conducted on a PTC-200 DNA engine (MJ Research): 95°C for 10 min; 45 cycles of 95°C for 20 s, 58°C for 20 s, and 72°C for 30 s; and a final extension at 72°C for 10 min. To purify PCR products, 5 *μ*L of the PCR products was treated with 1 *μ*L of ExoSAP-IT (catalogue number 78201; USB, Cleveland, OH, USA) at 37°C for 45 min. After that, the enzyme was inactivated by incubation at 80°C for 15 min.

We used twenty-two single base extension (SBE) primers recommended by Lee et al. [2]. SBE reactions were carried out using a SNaPshot Kit (Applied Biosystems, USA) according to the manufacturer's instructions. Thermal cycling conditions for SBE were as follows: denaturation at 96°C for 10 sec; annealing at 50°C for 5 sec; extension at 60°C for 30 sec. SBE was performed using a PTC-200 DNA Engine (Bio-Rad Laboratories, Hercules, CA). For postextension treatment, reaction mixtures were mixed with 1.0 unit of shrimp alkaline phosphatase (SAP), incubated at 37°C for 45 min, and followed by heat inactivation at 80°C for 15 min. The reactants were analyzed by an ABI PRISM 3100 Genetic Analyzer (Applied Biosystems, USA), using GeneMapper ID software, v3.2.1 (Applied Biosystems, USA). SNP scoring at each locus was confirmed by sequencing two samples for each of the observed alleles.

We also did direct sequencing of mtDNA control region of the samples. By sequencing of hypervariable regions I, II, and III, we could get haplotype of the bones and further determined haplogroups of them. The results could be compared with haplogroup determination by coding region SNP analysis on the same samples. Briefly, after quantification was done by NanoDrop ND-1000 Spectrophotometer (Thermo Fisher Scientific, MA, USA), 40 ng of aDNA was mixed with premix containing 1X Ampli*Taq* Gold 360 Master Mix (Life Technologies, USA) and 10 pmol of each primer (Integrated DNA Technology, USA). PCR conditions used in this study were as follows: predenaturation at 94°C for 10 min; 45 cycles of denaturation at 94°C for 30 sec; annealing at 50°C for 30 sec; extension at 72°C for 30 sec; final extension at 72°C for 10 min. PCR amplification was performed using a PTC-200 DNA Engine (Bio-Rad Laboratories, Hercules, CA). Primer sets used for this study were as follows: for 267-bp HV1A, F15971 (5′-TTA ACT CCA CCA TTA GCA CC-3′) and R16237 (5′-TGT GTG ATA GTT GAG GGT TG-3′); for 267-bp HV1B, F16144 (5′-TGA CCA CCT GTA GTA CAT AA-3′) and R16410 (5′-GAG GAT GGT GGT CAA GGG AC-3′); for 226-bp HV2A, F015 (5′-CAC CCT ATT AAC CAC TCA CG-3′) and R240 (5′-TAT TAT TAT GTC CTA CAA GCA-3′); for 235-bp HV2B, F155 (5′-CTA TTA TTT ATC GCA CCT-3′) and R389 (5′-CTG GTT AGG CTG GTG TTA GG-3′); for 167-bp HV3, F403 (5′-TCT TTT GGC GGT ATG CAC TTT-3′) and R569 (5′-GGT GTA TTT GGG GTT TGG TTG-3′) [26].

The PCR products were separated on 2.5% agarose gel, stained with ethidium bromide, and then isolated using a Qiagen gel extraction kit (Qiagen, Germany). The sequencing of each amplicon was performed by ABI Prism 3100 Genetic Analyzer (Applied Biosystems, USA), using ABI Prism BigDye Terminator Cycle Sequencing Ready Reaction Kit (Applied Biosystems, USA). The obtained DNA sequences were compared with the revised Cambridge Reference Sequence (rCRS; accession number: NC_012920), to identify the sequence differences between them. The resultant control region mutation motifs were imported into program mtDNAmanager (http://mtmanager.yonsei.ac.kr/), with which most Korean mtDNA haplotypes can be automatically classified into East Asian mtDNA haplogroups and their subhaplogroups [27], or another web-based program for mtDNA haplogroup analysis (http://dna.jameslick.com/mthap/) [28, 29].

In order to guard against any modern DNA contamination of ancient samples, the mtDNA profiles of all of the researchers involved in this study were determined (with the permission of the Institutional Review Board of Seoul National University, H-0909-049-295). They were then compared with the mtDNA profiles from the Joseon skeletons to rule out the possibility of modern DNA contamination.

## 3. Results

**Figure 1:**
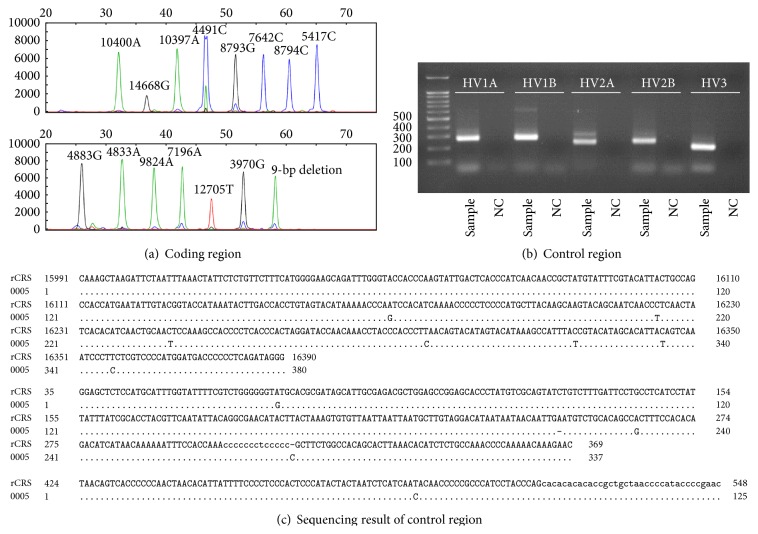
Analysis on sample number 005. (a) SNP analysis of mtDNA coding region by a SNaPshot Kit. ((b), (c)) Direct sequencing analysis on mtDNA control region. (b) Electrophoresis of PCR amplicons. (c) Direct sequencing result. rCRS, revised Cambridge Reference Sequence. Haplogroups (M8) determined by coding region SNP analysis (a) and control region direct sequencing ((b)/(c)) are the same for this case.

**Figure 2:**
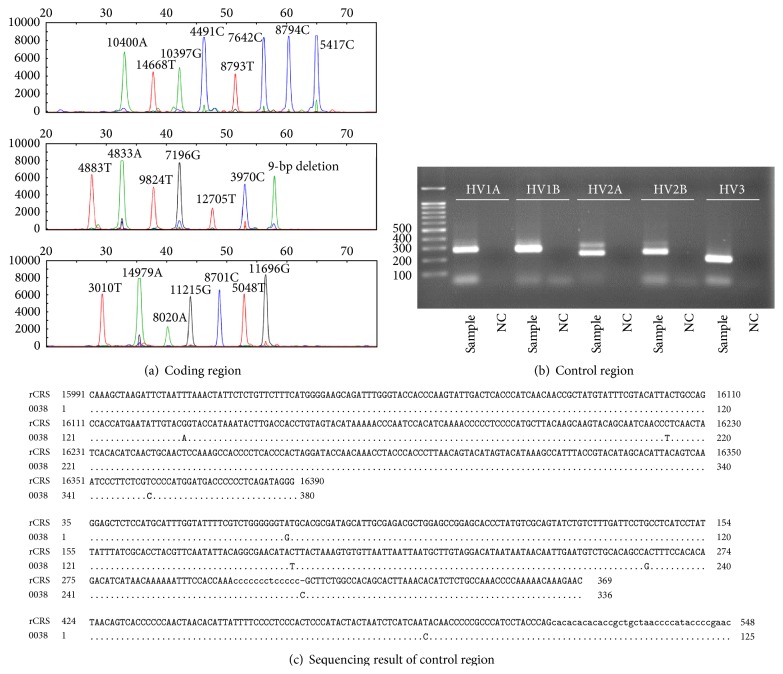
Analysis on sample number 038. (a) SNP analysis of mtDNA coding region by a SNaPshot Kit. ((b), (c)) Direct sequencing analysis on mtDNA control region. (b) Electrophoresis of PCR amplicons. (c) Direct sequencing result. rCRS, revised Cambridge Reference Sequence. Although mtDNA haplogroup expected by coding region SNP analysis could fall successfully within the same haplogroup that control region sequencing could make, haplogroup subclade made by SNP analysis (D4b) was not as successful as seen in control region sequencing (D4b2b).

The sex and age of the samples determined in this study are summarized in Table 1. By direct sequencing of mtDNA control region, every Joseon skeleton could be assigned to relevant existing haplo- or subhaplogroups of mtDNA. In multiplex PCR analyses to detect 21 SNP markers in mtDNA coding region, eight (multiplex I) and seven (multiplexes II and III) primer extension peaks for different haplogroups could be observed. Most samples exhibited no missing or extra assignment peaks in the results (Figures 1 and 2).

When the coding region SNP typing data were further compared with control region direct sequencing results, well-matching patterns can be observed between the outcomes of two methods. The mtDNA haplogroup expected by coding region SNP could fall successfully within the same haplogroup that control region sequencing could make (Table 1, Figure 1). However, as far as haplogroup subclades are concerned, the results of direct sequencing on control region mutation motif were far better than SNP marker analysis on coding region. The haplogroup subclades of the cases numbers 024, 034, 036, 038, 040, 045, 100, and 238 were much successfully determined in direct sequencing of control region than in coding region SNP analysis (Table 1, Figure 2).

The absence of modern DNA contamination could be confirmed by the comparison of mtDNA haplotypes obtained from the current Joseon skeleton and participating researchers' samples. As we did not see any identical sequence between them (Table 1), mtDNA obtained from Joseon samples should have been the endogenous DNA of the ancient people but not the outcome of modern DNA contamination from researchers.

## 4. Discussion

Coding region SNP analysis attracts forensic scientists' interest because it is a time-, cost-, and target DNA-saving method for mtDNA analysis of degraded samples [2, 30–35]. Multiplex PCR system for coding region SNP used in this study was originally designed for the detection of major haplogroups of forensic samples from East Asia. In the study, the triple multiplex system on coding region SNP markers was proven to be very useful for analysis on forensic materials [2]. Briefly, when they tried to do the coding region SNP-based mtDNA analysis on the long bone or molar samples from about 50-year-old skeletal remains of war victims in the Korean War (1950–1953), mtDNA haplogroup could be determined successfully, even with a limited volume of degraded DNA [2]. In fact, as the triple multiplex system makes very high success rate in haplogroup determination, the combined consideration of coding region SNP markers and control region polymorphism can become a very useful tool for the genetic analysis of degraded samples collected from East Asian populations.

This technique is also very suggestive for anthropologists who deal with several hundred- to thousand-year-old samples from archaeological sites. Most of the archaeological human bones were maintained under the worst preservation conditions for a long while. To make matters worse, it is very hard for researchers to get authentic outcomes from the ancient samples because only the limited volume of the samples can be allowed for the analysis of archaeologically important cases. We therefore admit that mtDNA analyses with highly degraded ancient samples are sometimes too risky because sequencing errors commonly occurred during analysis, and they could not be corrected easily by a due course of repeated experiments with a sufficient amount of samples.

In this regard, if another time-, cost-, and sample-saving mtDNA analysis could be also established for the assignment of ancient skeleton samples to relevant existing haplogroups, it will become a convenient method indeed for counterchecking the possible errors hidden in the conventional mtDNA sequencing. Our current results showed that most haplogroup results determined by coding region SNP analysis on Joseon archaeological samples can fall successfully within the same haplogroups that were decided by control region sequencing analysis. In fact, the results confirm coding region SNP analysis' error-screening role in the mtDNA haplogroup determination even for the ancient samples.

However, as for the current ancient samples, we must also admit some technical limits of haplogroup determination based on coding region SNP analysis. Briefly, a coding region SNP analysis on a few samples did not show the subhaplogroup results as completely as observed in the analysis of control region mutation motifs. This means that multiplex SNP analysis could not completely replace the conventional control region mtDNA sequencing, at least for the ancient cases from archaeological fields in South Korea. Even so, considering coding region SNP analysis' superb potential for reconfirmation of haplogroups determined by mtDNA control region sequencing in time-, cost-, and target DNA-saving manner, the use of this method can be expedient for making mtDNA haplogroup determination of archaeological samples much authentic.

## 5. Conclusion

Obtaining authentic mtDNA outcomes from archaeological bone samples still remains a significant challenge to concerned researchers. The identification of possible errors in conventional sequencing as quickly as possible is thus significant for authentic mtDNA analysis of archaeologically obtained samples. In this study, we can show that mtDNA haplogroup determination can also be successfully carried out by coding region SNP analysis, in a time-, cost-, and target DNA-saving manner. Although the mtDNA subhaplogroups could not be determined by the coding region SNP analysis as completely seen in the control region sequencing, the former can be used as good supplementary to the latter, for making the mtDNA typing of archaeological human bones much authentic.

## Figures and Tables

**Table 1:** Sequencing analysis result of mtDNA coding and control region.

Subject	Sex	Age	Hypervariable region			Haplogroup (control region)	Haplogroup (coding region)
			HVI (15991–16390)	HVII (034–369)	HVIII (423–548)		
004	Male	Old	16223T, 16235G, 16290T	73G, 235G, 263G, 315.1C	rCRS	A	A
005	Female	Young	16171G, 16223T, 16248T, 16298C, 16327T, 16344T, 16357C	73G, 248d, 263G, 315.1C	489C	M8	M8
024	Male	Middle	16129A, 16223T, 16309G, 16362C	73G, 152C, 263G, 309.1C, 315.1C	489C, 533G	D4a1b1	D4a
034	Male	Old	16189C, 16232A, 16249C, 16304C, 16311C, 16344T	73G, 263G, 310C	489C	M1a3b1	M
036	Male	Old	16150T, 16183C, 16185T, 16189C	73G, 151T, 197G, 263G, 315.1C	546G	B4d3	B
038	Male	Old	16129A, 16223T, 16362C	73G, 194T, 263G, 315.1C	489C	D4b2b	D4b
040	Male	Middle	16188.1C, 16193.1C, 16223T, 16311C	73G, 263G, 309.1C, 315.1C	489C	M4′′67	M
045	Male	Old	16223T, 16234T, 16311C, 16316G, 16362C	73G, 263G, 309.1C, 315.1C	489C	M9a1a1c1b1	M9
100	Male	Middle	16111T, 16229A, 16257A, 16261T, 16362C	73G, 150T, 263G, 309.2C, 315.1C	rCRS	N9a	N9
225	Male	Middle	16209C, 16223T, 16362C	73G, 195C, 234G, 263G, 315.1C	489C	D4	D4
238	Female	Old	16223T, 16295T, 16319A	73G, 146C, 263G, 309.1C, 315.1C	489C, 513d, 514d	M7c1a3	M7
Researcher 1	—	—	16172C, 16174T, 16223T, 16362C	73G, 263G, 309.1C, 315.1C	—	D4g	—
Researcher 2	—	—	16183C, 16189C, 16220C, 16254G, 16298C, 16362C	73G, 248d, 263G, 310.1C	—	F3b	—
Researcher 3	—	—	16129A, 16182C, 16183C, 16189C, 16232A, 16249C, 16304C, 16311C, 16344T	73G, 152C, 248d, 263G, 310.1C	—	D6c	—

## References

[1] Butler J. M. (2005). *Forensic DNA Typing: Biology and Technology behind STR Markers*.

[2] Lee H. Y., Yoo J.-E., Park M. J., Chung U., Kim C.-Y., Shin K.-J. (2006). East Asian mtDNA haplogroup determination in Koreans: haplogroup-level coding region SNP analysis and subhaplogroup-level control region sequence analysis. *Electrophoresis*.

[3] Kim M. J., Oh C. S., Lee I. S. (2008). Human mummified brain from a medieval tomb with lime-soil mixture barrier of the Joseon Dynasty, Korea. *International Journal of Osteoarchaeology*.

[4] Kim D. K., Lee I. S., Kim W.-L. (2011). Possible rheumatoid arthritis found in the human skeleton collected from the tomb of Joseon Dynasty, Korea, dating back to the 1700s AD. *International Journal of Osteoarchaeology*.

[5] Kim Y. S., Oh C. S., Lee S. J., Park J. B., Kim M. J., Shin D. H. (2011). Sex determination of Joseon people skeletons based on anatomical, cultural and molecular biological clues. *Annals of Anatomy*.

[6] Kim D. K., Kim M. J., Kim Y. (2013). Long bone fractures identified in the Joseon Dynasty human skeletons of Korea. *Anatomy & Cell Biology*.

[7] Han S. S., Baek K.-W., Shin M. H. (2010). Dental caries prevalence of medieval Korean people. *Archives of Oral Biology*.

[8] Shin D. H., Oh C. S., Kim Y.-S., Hwang Y.-I. (2012). Ancient-to-modern secular changes in Korean stature. *American Journal of Physical Anthropology*.

[9] Beom J., Woo E. J., Lee I. S. (2014). Harris lines observed in human skeletons of Joseon Dynasty, Korea. *Anatomy & Cell Biology*.

[10] Phenice T. W. (1969). A newly developed visual method of sexing the os pubis. *American Journal of Physical Anthropology*.

[11] Krogman W. M., Iscan M. Y. (1986). *The Human Skeleton in Forensic Medicine*.

[12] Buikstra J. E., Ubelaker D. H. (1994). *Standards for Data Collection from Human Skeletal Remains*.

[13] Ubelaker D. H. (1999). *Human Skeletal Remains: Excavation, Analysis, Interpretation*.

[14] Lovejoy C. O., Meindl R. S., Pryzbeck T. R., Mensforth R. P. (1985). Chronological metamorphosis of the auricular surface of the ilium: a new method for the determination of adult skeletal age at death. *American Journal of Physical Anthropology*.

[15] O'Rourke D. H., Hayes M. G., Carlyle S. W. (2000). Ancient DNA studies in physical anthropology. *Annual Review of Anthropology*.

[16] Rohland N., Hofreiter M. (2007). Ancient DNA extraction from bones and teeth. *Nature Protocols*.

[17] Yang D. Y., Eng B., Waye J. S., Dudar J. C., Saunders S. R. (1998). Improved DNA extraction from ancient bones using silica-based spin columns. *The American Journal of Physical Anthropology*.

[18] Casas M. J., Hagelberg E., Fregel R., Larruga J. M., González A. M. (2006). Human mitochondrial DNA diversity in an archaeological site in *al-Andalus*: genetic impact of migrations from North Africa in Medieval Spain. *American Journal of Physical Anthropology*.

[19] Blow M. J., Zhang T., Woyke T. (2008). Identification of ancient remains through genomic sequencing. *Genome Research*.

[20] Calvignac S., Hughes S., Tougard C. (2008). Ancient DNA evidence for the loss of a highly divergent brown bear clade during historical times. *Molecular Ecology*.

[21] Hofreiter M., Serre D., Poinar H. N., Kuch M., Pääbo S. (2001). Ancient DNA. *Nature Reviews Genetics*.

[22] Kivisild T., Tolk H. V., Parik J. (2002). The emerging limbs and twigs of the East Asian mtDNA tree. *Molecular Biology and Evolution*.

[23] Kong Q.-P., Yao Y.-G., Liu M. (2003). Mitochondrial DNA sequence polymorphisms of five ethnic populations from northern China. *Human Genetics*.

[24] Kong Q.-P., Bandelt H.-J., Sun C. (2006). Updating the East Asian mtDNA phylogeny: a prerequisite for the identification of pathogenic mutations. *Human Molecular Genetics*.

[25] Tanaka M., Cabrera V. M., González A. M. (2004). Mitochondrial genome variation in Eastern Asia and the peopling of Japan. *Genome Research*.

[26] Holland M. M., Huffine E. F. (2001). Molecular analysis of the human mitochondrial DNA control region for forensic identity testing. *Current Protocols in Human Genetics*.

[27] Lee H. Y., Song I., Ha E., Cho S.-B., Yang W. I., Shin K.-J. (2008). mtDNAmanager: a Web-based tool for the management and quality analysis of mitochondrial DNA control-region sequences. *BMC bioinformatics*.

[28] Aulicino E. D. (2013). *Genetic Genealogy: The Basics and Beyond*.

[29] Kennett D. (2012). *DNA and Social Networking: A Guide to Genealogy in the Twenty-First Century*.

[30] Bandelt H. J., Lahermo P., Richards M., Macaulay V. (2001). Detecting errors in mtDNA data by phylogenetic analysis. *International Journal of Legal Medicine*.

[31] Bandelt H.-J., Quintana-Murci L., Salas A., Macaulay V. (2002). The fingerprint of phantom mutations in mitochondrial DNA data. *The American Journal of Human Genetics*.

[32] Bandelt H. J., Salas A., Lutz-Bonengel S. (2004). Artificial recombination in forensic mtDNA population databases. *International Journal of Legal Medicine*.

[33] Forster P. (2003). To err is human. *Annals of Human Genetics*.

[34] Yao Y. G., Bravi C. M., Bandelt H. J. (2004). A call for mtDNA data quality control in forensic science. *Forensic Science International*.

[35] Salas A., Carracedo A., Macaulay V., Richards M., Bandelt H.-J. (2005). A practical guide to mitochondrial DNA error prevention in clinical, forensic, and population genetics. *Biochemical and Biophysical Research Communications*.

